# Comparing the Performance of Two Radiomic Models to Predict Progression and Progression Speed of White Matter Hyperintensities

**DOI:** 10.3389/fninf.2021.789295

**Published:** 2021-12-01

**Authors:** Yuan Shao, Jingru Ruan, Yuyun Xu, Zhenyu Shu, Xiaodong He

**Affiliations:** ^1^Department of Radiology, Zhejiang Provincial People’s Hospital, Affiliated People’s Hospital of Hangzhou Medical College, Hangzhou, China; ^2^Bengbu Medical College, Bengbu, China

**Keywords:** white matter, penumbra, radiomics, magnetic resonance imaging, texture analysis

## Abstract

**Purpose:** The aim of this study was to compare two radiomic models in predicting the progression of white matter hyperintensity (WMH) and the speed of progression from conventional magnetic resonance images.

**Methods:** In this study, 232 people were retrospectively analyzed at Medical Center A (training and testing groups) and Medical Center B (external validation group). A visual rating scale was used to divide all patients into WMH progression and non-progression groups. Two regions of interest (ROIs)—ROI whole-brain white matter (WBWM) and ROI WMH penumbra (WMHp)—were segmented from the baseline image. For predicting WMH progression, logistic regression was applied to create radiomic models in the two ROIs. Then, age, sex, clinical course, vascular risk factors, and imaging factors were incorporated into a stepwise regression analysis to construct the combined diagnosis model. Finally, the presence of a correlation between radiomic findings and the speed of progression was analyzed.

**Results:** The area under the curve (AUC) was higher for the WMHp-based radiomic model than the WBWM-based radiomic model in training, testing, and validation groups (0.791, 0.768, and 0.767 vs. 0.725, 0.693, and 0.691, respectively). The WBWM-based combined model was established by combining age, hypertension, and rad-score of the ROI WBWM. Also, the WMHp-based combined model is built by combining the age and rad-score of the ROI WMHp. Compared with the WBWM-based model (AUC = 0.779, 0.716, 0.673 in training, testing, and validation groups, respectively), the WMHp-based combined model has higher diagnostic efficiency and better generalization ability (AUC = 0.793, 0.774, 0.777 in training, testing, and validation groups, respectively). The speed of WMH progression was related to the rad-score from ROI WMHp (*r* = 0.49) but not from ROI WBWM.

**Conclusion:** The heterogeneity of the penumbra could help identify the individuals at high risk of WMH progression and the rad-score of it was correlated with the speed of progression.

## Introduction

White matter hyperintensity (WMH) is a common feature found in the periventricular and deep white matter of the elderly (Longstreth et al., 2005). Maillard et al. (2014) reported that the abnormal regions are perceived as larger on diffusion tensor imaging (DTI) than on fluid-attenuated inversion recovery (FLAIR) images and proposed the concept of “structural penumbra.” A prospective study reported that 80% of WMH progression appears as direct extensions of preexisting lesions rather than new, scattered lesions (de Groot et al., 2013). Also, this study revealed the pattern of WMH progression, which was found to extend from the focus to the penumbra. Our study was aimed at comparing the performance of two radiomic models to predict the progression and progression speed of white matter hyperintensities.

Previous studies have suggested that the FLAIR intensity of a single voxel assists DTI in predicting WMH progression, indicating that the intensity of an individual voxel in conventional Magnetic resonance imaging (MRI) possibly contains influential information regarding the integrity of the white matter (Maillard et al., 2013). Radiomics is a relatively new field that could reveal changes in the microstructure and its regularity by extracting the intensity value of a single voxel and analyzing its relationship with the intensity value of neighboring voxels and its position within the brain (Yip and Aerts, 2016). Our previous research also found that radiomic findings could reflect the heterogeneity and complexity of the white matter, which may result from less uniform MRI signal intensities caused by reduced myelin content or increased water content (Shao et al., 2018; Shu Z. et al., 2020; Shu Z. Y. et al., 2020). Tozer et al. (2018) showed that the texture of the whole-brain white matter (WBWM) was moderately correlated with global cognition and executive dysfunction, and they may be less sensitive than DTI parameters in predicting cognitive decline (Tozer et al., 2018). We thought that considering the WBWM as the region of interest (ROI) may reduce the predictive power as it would contain more normal tissues. However, the heterogeneity and complexity of the penumbra may be more representative of the lesions. To our knowledge, there have been no studies comparing the predictive power of the WBWM and penumbra.

This study was aimed at investigating whether the heterogeneity of the penumbra was more obvious than that of WBWM in identifying high-risk patients. Furthermore, we want to explore whether a correlation exists between radiomic findings and the speed of progression.

## Materials and Methods

### Subjects

This research was approved by the ethics committee, and the need of obtaining informed consent of patients was waived owing to the retrospective design of the study.

**FIGURE 1:**
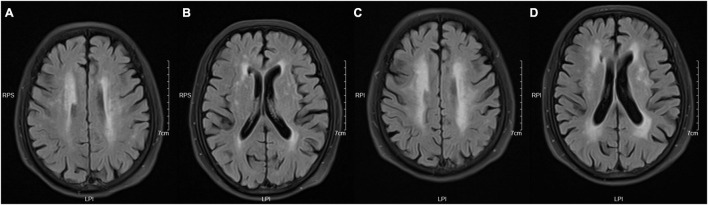
Take the periventricular score as an example. These images were obtained from a 73-year-old woman with a history of hypertension and diabetes. Panels **(A,B)** are baseline images, and **(C,D)** are follow-up images. These images show enlargement of white matter hyperintensity (WMH) in frontal caps, lateral bands, and occipital caps. So, the periventricular score for this patient is +3.

Magnetic resonance imaging data of 152 patients from Medical Center A (ZPP hospital) and 80 patients from Medical Center B (LSP hospital) were collected in this study. The labeled names of all patients of the Medical Center A dataset were listed in alphabetical order and divided into two sets: the division formed a training set (*n* = 105) and a testing set (*n* = 47) in the ratio of 7:3. The database of Medical Center B patients was used as an external validation dataset. Prins et al. (2004) proposed a visual rating scale in 2004; based on this scale, all patients were divided into WMH progression group (*n* = 57 in A and *n* = 31 in B) and non-progression group (*n* = 95 in A and *n* = 49 in B). Periventricular WMH (PWMH) and deep WMH (DWMH) were compared independently. PWMH was defined as WMH within 10 mm from the ventricle surface. WMH away from the ventricle surface 10 mm is defined as DWMH. Scores of −3 to +3 were given according to the progression or decrease in PWMH in the former horn, body, and posterior horn, as shown in Figure 1. Scores of −4 to +4 were given according to the progression or decrease in DWMH in different brain regions. WMH progression was defined when the total score was ≥1. WMHs were graded, and their volume was quantified using FLAIR images. Clinical information on various aspects, such as vascular risk factors, clinical course, age, and sex, was obtained from the medical records of the picture archiving and communications system.

**FIGURE 2:**
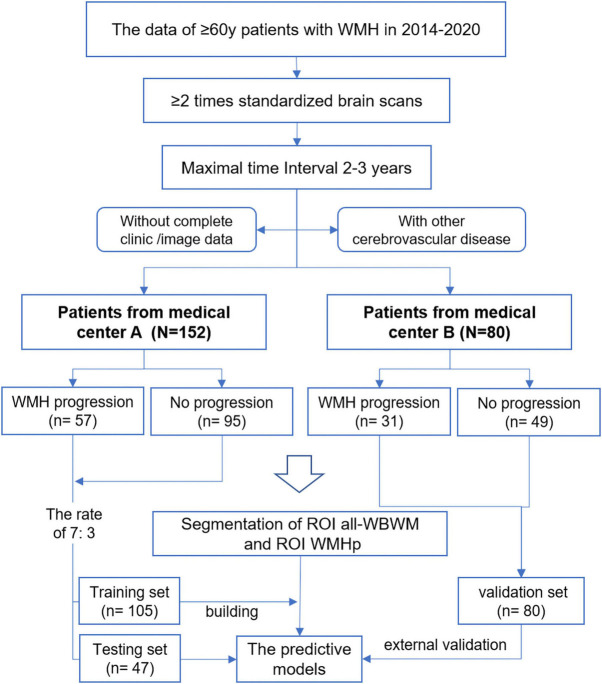
Flowchart of this study.

**FIGURE 3:**
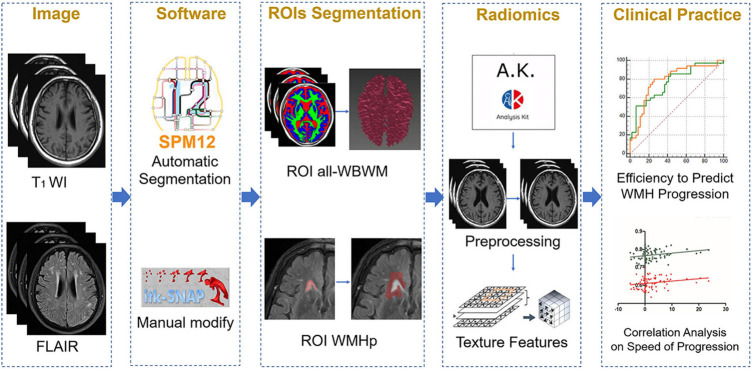
The overall outline of the radiomic procedure used in the current work, including image selection, region of interest (ROI) segmentation, model fitting, and clinical application.

We included patients who (1) had a clinical diagnosis of minor strokes or transient ischemic attacks, (2) underwent more than two MRI examinations on the same machine within an interval of 2–3 years, (3) were older than 60 years at the first examination, and (4) had visible WMH at baseline. We excluded patients who (1) had acute vascular lesions, such as ischemic stroke (except for lacunar infarction) or intracranial hemorrhage; (2) had non-vascular white matter lesions, such as immunologic demyelination, metabolic encephalopathy, poisoning, and infection; (3) had other intracranial lesions, including Alzheimer’s disease, Parkinson’s disease, craniocerebral trauma, or tumor; (4) had incomplete clinical data; (5) had incomplete imaging data; and (6) had imaging data with motion or machine artifacts. Figures 2, 3 show the flowchart summarizing participant recruitment and building radiomic models.

### Magnetic Resonance Imaging Acquisition

Brain MRI scans in medical centers A and B were performed using a 3.0 T MRI scanner with an eight-channel head coil (Discovery MR 750, GE Healthcare, Chicago, IL, United States). The routine sequences were as follows: axial T_1_WI, T_2_WI, FLAIR, and DWI. We used axial FLAIR to observe and segment the identified WMH based on the following parameters: TR = 9,000 ms, TE = 120 ms, field of view (FOV) = 220 mm × 220 mm, matrix = 256 × 256, and section thickness = 5 mm, and inter-slice gap = 1.5 mm. We used T_1_WI for segmenting the white matter with TR = 1,750 ms, TE = 24 ms, FOV = 220 mm × 220 mm, section thickness = 5 mm, and inter-slice gap = 1.5 mm. T_2_WI: TR = 9,823 ms, TE = 101 ms, FOV = 220 mm × 220 mm, section thickness = 5 mm, and inter-slice gap = 1.5 mm. DWI: TR = 3,071 ms, TE = minimum, *b* = 0, 1,000 s/mm^2^, FOV = 220 mm × 220 mm, inter-slice gap = 1.5 mm, and section thickness = 5 mm.

### Image Preprocessing

All MRI scan sequences were converted to NIfTI files (.nii.gz) and preprocessed for normalization. Different imaging sequences were co-registered to the same anatomical template; then, they were interpolated to the same resolution (1 mm × 1 mm × 1 mm) and skull-stripped (Rohlfing et al., 2010; Bakas et al., 2017). The noise in the images was reduced using Gaussian filtering; then, for reducing external interference factors, magnetic field migration correction was performed. Then, by downsampling each image into 32 bins, the image grayscale intensity level was discretized and normalized for noise reduction. Given these fixed bins values and numbers, the grayscale range of the image is divided into equally spaced intervals. Thus, the grayscale values reflected the size and intensity resolution of the discretized bins (i.e., there are four sized bins per grayscale).

### Segmentation of Region of Interests

#### Region of Interest Whole-Brain White Matter

After spatially normalizing the MRI images to a universal coordinate system, the gray matter, white matter, and cerebrospinal fluid (GM/WM/CSF) of the whole brain were automatically segmented in T_1_WI using the Statistical Parametric Maps 12 (SPM12) toolbox.^1^ The automatically segmented WM was treated as the ROI WBWM.

#### Region of Interest White Matter Hyperintensity Penumbra

The WMH was segmented automatically using FLAIR and T_1_WI. Maillard et al. (2014) defined the WMH penumbra as the 5 mm area surrounding the WMH. We used the AGK software (Artificial-Intelligent Radio-Genomics Kits; GE Healthcare, Chicago, IL, United States) to automatically expand the WMH region by 5 mm. Then, the sulcus and gyrus were manually removed by two experienced neuroradiologists using the ITK-SNAP software.^2^ Supplementary Figure 1 shows a diagram describing this ROI segmentation approach. All segmentations were visually checked for segmentation errors and artifacts.

### Extraction of Radiomic Features

All MRI images and ROIs were imported into the AGK software to extract radiomic features. The radiomic features were calculated, including histogram, texture, form factor, gray-level co-occurrence matrix (GLCM), run-length matrix (RLM), and gray-level size zone matrix (GLSZM). The extracted texture features were standardized, which removed the unit limits of the data of each feature and converted it into a dimensionless pure value. This allowed the indexes of different units or orders to be compared and weighted. For details, see Supplementary Materials.

### Construction and Assessment of the Radiomic Models

Based on the training set, we performed analysis of variance (ANOVA) of the extracted features. For feature dimensionality reduction, the analysis of ANOVA + Mann–Whitney *U*-test, correlation analysis, and gradient boosting decision tree were sequentially performed. See Supplementary Materials for details. The five machine learning methods, namely Bayes, the random forest, the logistic regression, the support vector machine classifiers (SVM), and the *k*-nearest neighbor (KNN), were used to build models, and the best modeling method was selected through comparison. The test set, the training set, and the external verification set were used to verify the predictive efficiency, including calibration efficiency, net value, and diagnostic accuracy, which were estimated using the calibration curve, the decision curve analysis (DCA), and receiver operating characteristic (ROC) curve.

### Interobserver and Intra-Observer Reproducibility

For eliminating the sulcus and gyrus, the ROI WMHp was first manually adjusted by the physician XDH. One month later, the physicians XDH and YS manually eliminated these again on 30 randomly selected subjects. The intra-observer correlation coefficient was calculated based on the first measurement of the physicians XDH and YS. The interobserver correlation coefficient was calculated based on the two measurements acquired by the physician XDH.

### Statistical Analysis

We performed our statistical analyses using SPSS 20.0 (IBM, Chicago, IL, United States). Comparisons of clinical and imaging characteristics were performed using a *T*-test, Mann–Whitney *U*-test, or chi-square test. Moreover, we performed univariate logistic regression analyses on each potential predictor variable associated with WMH progression, including age, sex, imaging factors, and vascular risk factors. Thereafter, to construct combined prediction models, factors with marginal significance (*P* < 0.1) in univariate logistic regression were included in multivariable logistic regression. The pairwise correlation among clinical features, the radiomic score (rad-score), and the speed of WMH progression were calculated using the Spearman’s analysis. The values of *P* ≤ 0.05 were considered to indicate statistical significance.

## Results

### Demographic and Clinical Characteristics

Table 1 shows the imaging features and demographic characteristics of the participants. In Medical Center A, the median age of patients with WMH progression was significantly higher than for those without WMH progression (74 years vs. 68 years, *P* = 0.004). In Medical Center B, the average age of patients was higher in the WMH progression group than in the non-progression group; however, the difference was not statistically significant (73.52 years vs. 71.65 years, *P* = 0.327). In both medical centers, the difference in the volume and speed of WMH progression was statistically significant between the two groups (all *P* < 0.05). We found no significant difference in imaging features and demographic characteristics between medical centers A and B (all *P* > 0.05).

**TABLE 1:** The demographic characteristics and imaging features of the participants in medical centers A and B.

	**Progression of WMH in A center *n* = 152**			**Progression of WMH in B center *n* = 80**			**A vs. B**
	**No (*n* = 95)**	**Yes (*n* = 57)**	***P***	**No (*n* = 49)**	**Yes (*n* = 31)**	***P***	***P***
Age (years)	68 (64–75)	74 (68–80)	0.004*	71.65 ± 8.92	73.52 ± 7.00	0.327	0.367*
Sex (male)	49 (51.58%)	26 (45.61%)	0.476	25 (51.02%)	15 (48.39%)	0.818	0.819
Hypertension	48 (50.53%)	37 (64.91%)	0.084	24 (48.98%)	13 (41.94%)	0.538	0.569
Diabetes	39 (41.05%)	20 (35.09%)	0.465	17 (34.69%)	13 (41.94%)	0.515	0.552
Hyperlipidemia	37 (38.95%)	25 (43.86%)	0.551	24 (48.98%)	15 (48.39%)	0.959	0.387
CHD	33 (34.74%)	23 (40.35%)	0.487	19 (38.78%)	9 (29.03%)	0.373	0.674
Smoking	38 (40.00%)	19 (33.33%)	0.411	22 (44.90%)	12 (38.71%)	0.585	0.360
Drinking	51 (53.68%)	28 (49.12%)	0.586	30 (61.22%)	17 (54.84%)	0.572	0.274
The Fazekas score of DWMH			0.414			0.736	0.771
1	17 (17.89%)	19 (33.33%)		15 (30.61%)	6 (19.35%)		
2	59 (62.11%)	23 (40.35%)		22 (44.90%)	23 (74.19%)		
3	19 (20.00%)	15 (26.32%)		12 (24.49%)	2 (6.45%)		
The Fazekas score of PWMH			0.629			0.952	0.779
1	18 (18.95%)	18 (31.58%)		13 (26.53%)	6 (19.35%)		
2	55 (57.89%)	22 (38.60%)		24 (48.98%)	20 (64.52%)		
3	22 (23.16%)	17 (29.82%)		12 (24.49%)	5 (16.13%)		
Volume of WMH at baseline (cm^3^)	16.40 ± 13.68	20.79 ± 17.87	0.114	16.59 ± 11.85	21.19 ± 11.54	0.091	0.389
Volume of WMH progress (cm^3^)	1.52 ± 4.70	3.51 ± 5.80	0.023	1.18 ± 2.68	2.90 ± 3.70	0.018	0.107
Interval time (d)	862.57 ± 106.80	881.98 ± 100.48	0.269	863.37 ± 89.99	860.48 ± 109.09	0.898	0.766
Speed of progress (mm^3^/d)	1.74 ± 5.43	4.00 ± 6.68	0.024	1.39 ± 3.23	3.56 ± 4.73	0.017	0.118

CHD, coronary heart disease; DWMH, deep white matter hyperintensity; PWMH, periventricular white matter hyperintensity. *Mann–Whitney *U*-test, Median, 25–75%.

### Building Radiomic Models to Predict White Matter Hyperintensity Progression

**FIGURE 4:**
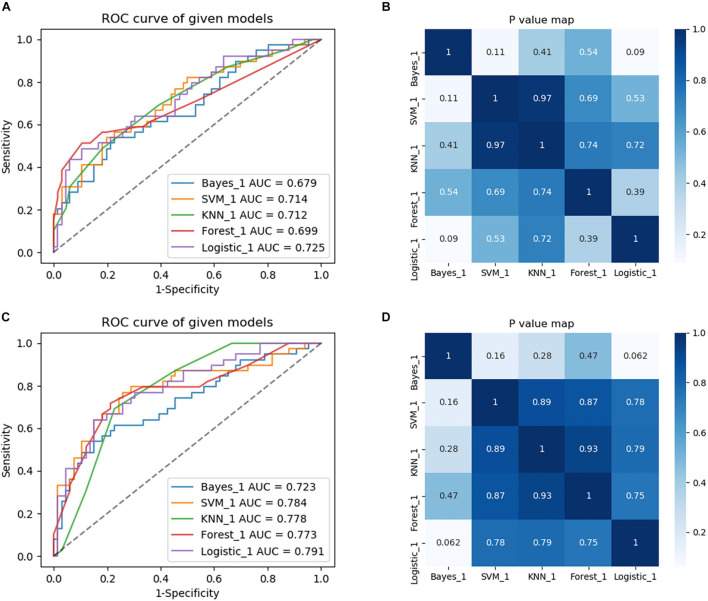
Comparison of diagnostic performance of five machine learning approaches and selection of the best machine learning approach to build models. **(A)** The region of interest (ROC) curves of the five machine learning methods in the training group of the ROI whole-brain white matter (WBWM). **(B)** A heatmap of *P*-values comparing diagnostic performance for five machine learning approaches in the ROI WBWM. **(C)** The ROC curves of the five machine learning methods in the training group of the ROI WMH penumbra (WMHp). **(D)** A heatmap of *P*-values comparing diagnostic performance for five machine learning approaches in ROI WMHp.

**FIGURE 5:**
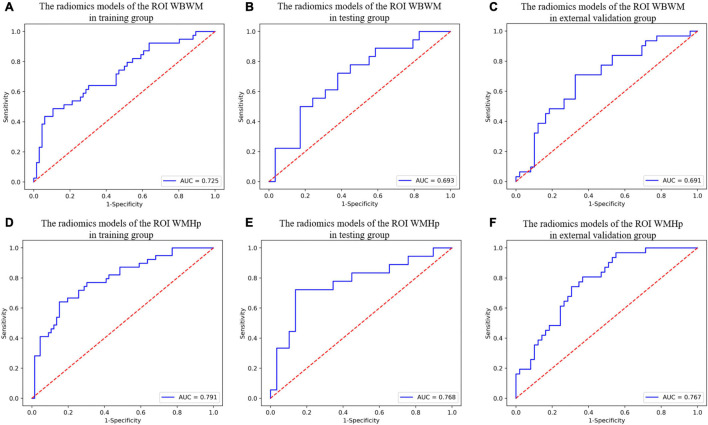
The diagnostic accuracy of radiomic models in different groups of two regions of interests (ROIs). **(A–C)** The ROC curves of the radiomic models of the ROI whole-brain white matter (WBWM) in the training group, testing group, and external validation group, respectively. **(D–F)** The ROC curves of the radiomic models of the ROI WMH penumbra (WMHp) in the training group, testing group, and external validation group, respectively.

**FIGURE 6:**
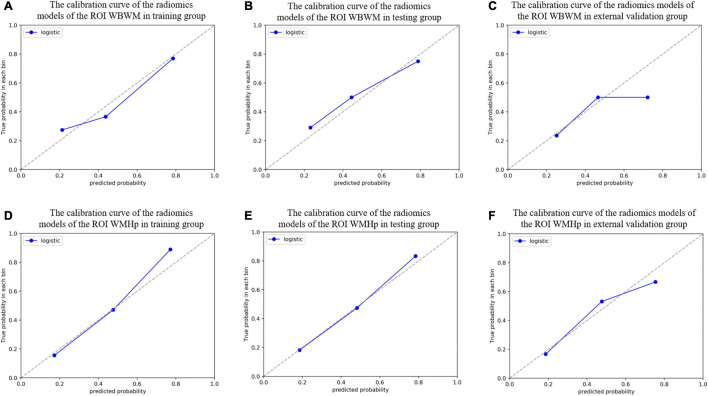
The calibration curve was used to describe the goodness-of-fit of radiomic models in different groups of two regions of interests (ROIs). **(A–C)** The radiomic models of the ROI whole-brain white matter (WBWM) in the training group, testing group, and external validation group, respectively. **(D–F)** The radiomic models of the ROI WMH penumbra (WMHp) in the training group, testing group, and external validation group, respectively. The Hosmer–Lemeshow test revealed good goodness-of-fit of all models (all *P* > 0.05).

**FIGURE 7:**
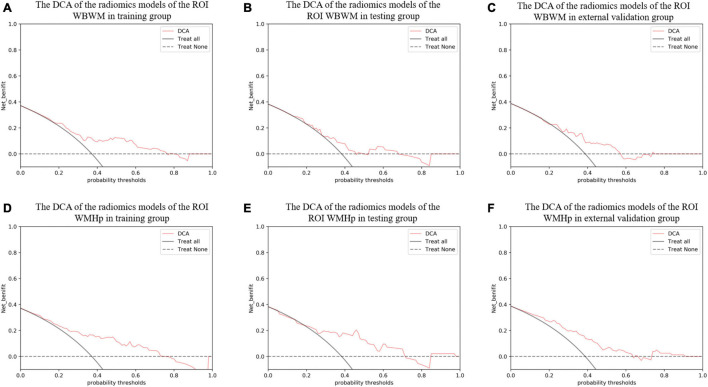
The decision curve analysis (DCA) curve was used to describe the net value of radiomic models in different groups of two regions of interests (ROIs). The *Y*-axis represents the net benefit. The pink line represents the radiomic model. The solid black line represents the hypothesis that all patients have white matter hyperintensity (WMH) progression. The black dotted line represents the hypothesis that no patients have WMH progression. The *X*-axis represents the threshold probability. The threshold probability is where the expected benefit of a treatment is equal to the expected benefit of avoiding the treatment. **(A–C)** The DCA of the radiomic models of the ROI whole-brain white matter (WBWM) in the training group, testing group, and external validation group, respectively. **(D–F)** The DCA of the radiomic models of the ROI WMH penumbra (WMHp) in the training group, testing group, and external validation group, respectively.

For constructing radiomic models after feature dimensionality reduction, the optimal features were selected, including 12 features in the ROI WMHp and 7 features in the ROI WBWM. The area under the curve (AUC) value was higher for the logistic regression model than for other machine learning methods. In line with this finding, the models were built using the logistic regression classifier (Figure 4). The rad-score was calculated using the formula for the features. The rad-score was found to be significantly different between the progression and non-progression groups in two ROIs (all *P* < 0.05; Table 2). Additional information about the formulas is shown in the Supplementary Material. The predictive efficacies (represented as the AUC) of the ROI WBWM were 0.725, 0.693, and 0.691 for the training group, testing group, and external validation group, respectively. Similarly, the predictive efficacies of the ROI WMHp were 0.791, 0.768, and 0.767 for the training group, testing group, and external validation group, respectively. Figures 5–7 show the diagnostic accuracy, the calibration efficiency, and the net value of models, which were evaluated using the ROC, the Hosmer–Lemeshow test, and DCA, respectively.

**TABLE 2:** Diagnostic accuracy of the rad-score in the training group, testing group, and external validation group.

	**WBWM**			**WMHp**		
	**Training group**	**Testing group**	**External validation**	**Training group**	**Testing group**	**External validation**
Rad-score of progression group	−0.91 ± 0.82	−0.94 ± 0.75	−0.80 ± 0.83	−1.08 ± 1.22	−1.13 ± 1.34	−1.41 ± 1.60
Rad-score of no-progression group	−0.07 ± 1.03	−1.25 ± 1.12	−0.27 ± 0.83	−0.01 ± 0.88	0.07 ± 1.33	0.04 ± 1.15
*P*	<0.001	0.046	0.006	<0.001	0.004	<0.001
AUC	0.725	0.693	0.691	0.791	0.768	0.767
Sensitivity	48.7%	72.2%	71.0%	64.1%	72.2%	80.6%
Specificity	89.5%	63.1%	67.3%	84.8%	86.2%	63.3%

### Building Combined Models to Predict White Matter Hyperintensity Progression

After stepwise logistic regression analysis, age, hypertension, and the rad-score of the ROI WBWM were the independent factors of WMH progression (Table 3). We used these factors to construct the combined model in WBWM. The AUCs were 0.779, 0.716, and 0.673 in the training group, testing group, and external validation group, respectively (Table 4). In addition, the combined model in WBWM was constructed using the age and the rad-score of the ROI WMHp (Table 3). The AUCs were 0.793, 0.774, and 0.777 in the training group, testing group, and external validation group, respectively (Table 4).

**TABLE 3:** Stepwise logistic regression analysis was performed to construct combined clinical and imaging models for predicting WMH progression.

	**Univariate logistic regression**		**Multivariate logistic regression**			
			**WMHp-based combined model**		**WBWM-based combined model**	
	**OR (95% CI)**	***P***	**OR (95% CI)**	***P***	**OR (95% CI)**	***P***
Age (years)	1.063 (1.016, 1.112)	0.008	1.077 (1.023, 1.133)	0.004	1.069 (1.015, 1.125)	0.011
Sex (male)	0.787 (0.408, 1.521)	0.477	/	/	/	/
Hypertension	1.811 (0.921, 3.563)	0.085	2.248 (1.029, 4.910)	0.042	1.647 (0.758, 3.581)	0.208
Diabetes	0.776 (0.393, 1.533)	0.465	/	/	/	/
Hyperlipidemia	1.225 (0.629, 2.384)	0.551	/	/	/	/
CHD	1.271 (0.646, 2.501)	0.488	/	/	/	/
Smoking	0.750 (0.377, 1.491)	0.412	/	/	/	/
Drinking	0.833 (0.432, 1.608)	0.586	/	/	/	/
Interval time	1.001 (0.998, 1.004)	0.655	/	/	/	/
Volume of WMH at baseline	1.018 (0.997, 1.04)	0.094	1.020 (0.999, 1.045)	0.103	1.013 (0.989, 1.038)	0.301
DWMH	0.82 (0.504, 1.333)	0.423	/	/	/	/
PWMH	0.886 (0.555, 1.415)	0.612	/	/	/	/
Rads-WBWM	2.251 (1.493, 3.395)	<0.001	2.779 (1.765, 4.376)	<0.001	/	/
Rads-WMHp	1.987 (1.51, 2.614)	<0.001	/	/	2.473 (1.639, 3.732)	<0.001

*Univariate logistic regression analysis was performed on each potential predictor of WMH progression, including age, sex, vascular risk factors, and imaging factors. Factors with marginal significance (*P* < 0.1) in the univariate logistic regression were included in the multivariate logistic regression to construct combined prediction models. Rads, rad-score.*

**TABLE 4:** The predictive efficacy of two joint models in training group, testing group, and validation group.

	**AUC**	**Sensitivity**	**Specificity**	**Positive prediction**	**Negative prediction**
**WBWM-based combined model**					
Training group	0.779	74.4%	77.3%	65.9%	83.6%
Testing group	0.716	72.2%	75.9%	65.0%	81.5%
Validation group	0.673	83.9%	55.1%	54.2%	84.4%
**WMHp-based combined model**					
Training group	0.793	56.4%	90.9%	78.6%	77.9%
Testing group	0.774	72.2%	75.9%	65.0%	81.5%
Validation group	0.777	90.3%	59.2%	58.3%	90.6%

### The Pairwise Correlation Among Clinical Risk Factors, Rad-Score, and Speed of Progression

**FIGURE 8:**
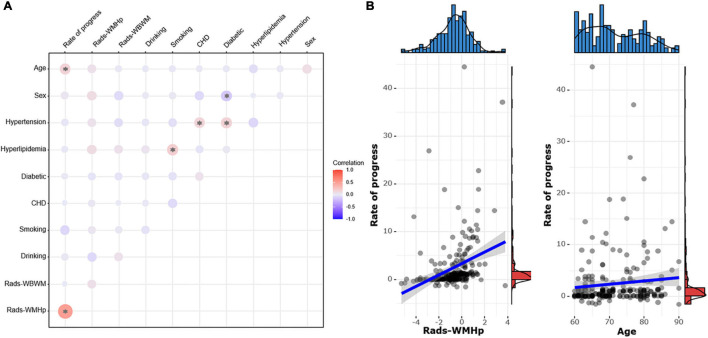
**(A)** The pairwise correlation heatmap among clinical risk factors, the rad-score, and the speed of white matter hyperintensity (WMH) progression. The color bar on the right represents the size of the correlation coefficient. The larger the circle in the figure, the higher the correlation. Asterisk indicates *P* < 0.05. CHD, coronary heart disease. **(B)** Scatter diagrams show the correlation between the speed of progress and the rad-score of WMH penumbra (WMHp) and age.

The speed of WMH progression was positively correlated with the rad-score from the ROI WMHp and age (*r* = 0.49 and *r* = 0.15, respectively). In addition, in our sample, we found a weak correlation between hypertension and coronary heart disease, hypertension and diabetes, and hyperlipidemia and smoking (*r* = 0.14, *r* = 0.16, and *r* = 0.18, respectively). Women were more likely to develop diabetes than men (*r* = −0.16), as shown in Figure 8.

### Interobserver and Intra-Observer Reproducibility

The intra- and interobserver correlation coefficients of segmenting the ROI WMHp were 0.846 and 0.885, respectively.

## Discussion

Our results revealed that the predictive efficiency was higher for the ROI WMHp-based radiomic model than the ROI WBWM-based radiomic model. Compared with the WBWM-based combined model, the WMHp-based combined model has higher diagnostic efficiency and better generalization ability. Another noteworthy result was that the speed of progression was related to the rad-score of the ROI WMHp but not to that of the ROI WBWM.

The efficiency of the WMHp-based radiomic model was higher than that of the WBWM-based radiomic model in predicting the WMH progression. This finding may be attributed to the fact that the evolutionary mechanism of WMH predominantly affects the foci and moves toward the periphery gradually (Maniega et al., 2015; Reginold et al., 2018; van Leijsen et al., 2018; Vangberg et al., 2019). The microstructure of the WMHp region has been suggested to be more heterogeneous and complex and more predictive of WMH progression. However, the predictive power of WBWM was reduced by the inclusion of more extensive normal tissues (Shu Z. et al., 2020). This result also suggests that in particular for clinical studies, the selection of ROIs by medical principles may be more important than the size of ROIs for diagnostic accuracy.

Moreover, we also found that the ROI WMHp-based combined model has higher better generalization ability in predicting the WMH progression. This model has been validated in an external cohort with good diagnostic efficiency, and age was the independent clinical factor that survived in the predictive model. The WMH is the main radiological feature of small vessel disease, with age as a confirmed risk factor (Grueter and Schulz, 2012; Sabisz et al., 2019). Besides, our findings suggested that hypertension could also be an established risk factor for the WMH progression in the ROI WBWM-based combined model. Hypertension would damage small blood vessel walls and increase blood–brain barrier permeability, thus aggravating white matter progression (Chen et al., 2019; Sabisz et al., 2019).

Previous studies using radiomics on the WMH progression ignored the effect of the interval time between examinations (Shu Z. et al., 2020; Shu Z. Y. et al., 2020). To avoid this lapse, we further studied the correlation between the speed of progression and the rad-score. Consequently, we found that the speed of the WMH progression to be related to the rad-score of only ROI WMHp and not of the ROI WBWM. The WMH progression follows the pattern of extending from the lesion to the adjacent regions, the heterogeneity, and the complexity of the penumbra was more representative of and correlated more strongly to the progression of the lesion (Maillard et al., 2014). However, the heterogeneity of WBWM was diluted by relatively more normal tissues, and it was not correlated with the speed of progression. We also found a mild correlation between age and the speed of progress, which corroborated previous reports (Schmidt et al., 2003; Grueter and Schulz, 2012).

This study has some limitations. First, the sample size was not large enough, so more cases need to be collected to verify the model. Second, semiautomatic segmentation of ROI WMHp was time-consuming than automatic delineation, which would reduce its clinical usefulness in future.

## Conclusion

Radiomic findings revealed that the damage of WMH extended further from the high-intensity area observed on conventional MRI sequences. The heterogeneity of the penumbra could identify the individuals at high risk of WMH progression and the rad-score of it was correlated with the speed of progression.

## Data Availability Statement

The raw data supporting the conclusions of this article will be made available by the authors, without undue reservation.

## Ethics Statement

The studies involving human participants were reviewed and approved by the Medical Ethics Committee of Zhejiang Provincial People’s Hospital. Written informed consent for participation was not required for this study in accordance with the national legislation and the institutional requirements.

## Author Contributions

XH designed this study and guided the experiment. YS and YX wrote this manuscript and participated in the whole experiment process. YS and ZS analyzed the data. All authors read and approved the final manuscript.

## Conflict of Interest

The authors declare that the research was conducted in the absence of any commercial or financial relationships that could be construed as a potential conflict of interest.

## Publisher’s Note

All claims expressed in this article are solely those of the authors and do not necessarily represent those of their affiliated organizations, or those of the publisher, the editors and the reviewers. Any product that may be evaluated in this article, or claim that may be made by its manufacturer, is not guaranteed or endorsed by the publisher.

## Footnotes

1
https://www.fil.ion.ucl.ac.uk/spm/software/spm12

2
http://www.itksnap.org/pmwiki/pmwiki.php

## Abbreviations

WMH: white matter hyperintensity
WBWM: whole-brain white matter
WMHp: white matter hyperintensity penumbra
ANOVA: analysis of variance
AUC: area under the curve
DTI: diffusion tensor imaging
FLAIR: fluid-attenuated inversion recovery
GLCM: gray-level co-occurrence matrix
GLSZM: Gray Level Size Zone Matrix
RLM: run-length matrix
ROC: receiver operating characteristic
ROI: regions of interest
FOV: field of view.
