# Supplementary material for: Comparing the Performance of Two Radiomic Models to Predict Progression and Progression Speed of White Matter Hyperintensities
Source: Front Neuroinform. 2021 Dec 1;15:789295. doi: 10.3389/fninf.2021.789295 (PMC8671609; doi:10.3389/fninf.2021.789295)
Supplement: Supplementary file 1 [file Data_Sheet_1.docx]

**Supplementary material**

Figure S1. The flowchart of drawing the WMHp ROI.


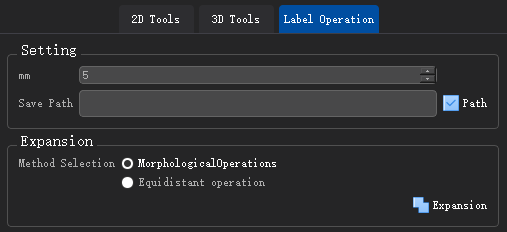

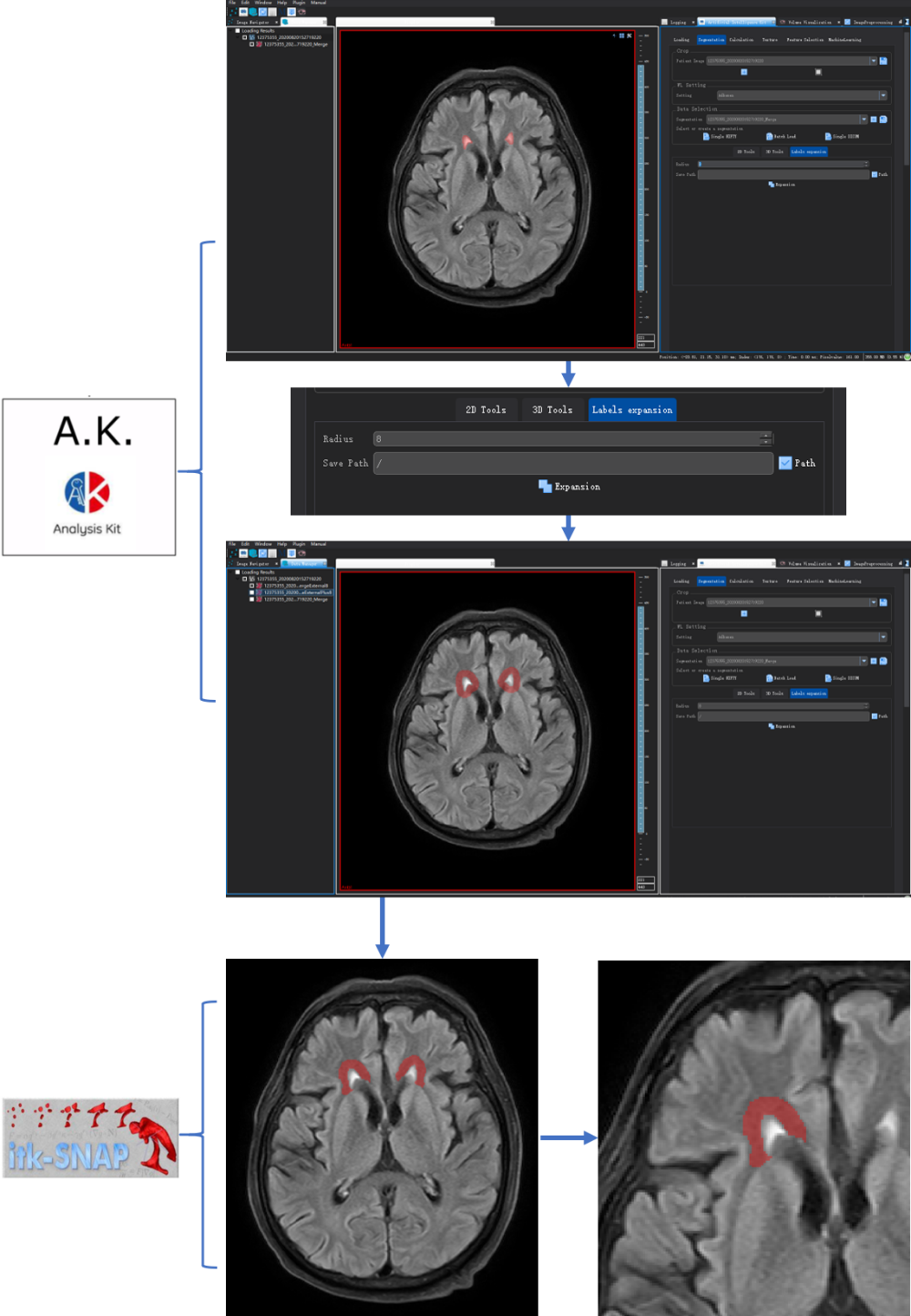


(http://www.itksnap.org/pmwiki/pmwiki.php)

(Artificial Intelligence Kit V3.0.0.R, GE Healthcare)

3. Initial WMHp ROIs were obtained

4. The sulcus and gyrus were manually eliminated using the ITK-SNAP software.

5. Finally, the region with the largest volume was selected as the WMHp ROI.

2. The WMH ROI was automatically expanded by 5mm.

1. Automatically segmented WMH ROIs were loaded into AK software.

Extracted texture features were standardized, which removed the unit limits of the data of each feature and converted it into a dimensionless pure value. This allowed the indexes of different units or orders to be compared and weighted. We used a z-score normalization to make the image intensities fit a standard normal distribution with $\mu\text{=0}$ and $\sigma\text{=1}$, where $\mu$ is the mean value of the images, and $\sigma$ is the standard deviation. The normalized values (also called z-scores) of the image intensities (*x*) were calculated as follows:

$$z=\frac{x-\mu}{\sigma}$$

After image z-score normalization, texture features were then extracted using the AK software.

Radiomics features included the histogram (42 features), Haralick (10 features), Formfactor (9 features), Gray-Level Co-occurrence Matrix (126 feature, GLCM), Run length matrix (180 features, RLM) and Gray Level Size Zone Matrix (11 features, GLSZM). The feature details are described in the table below.


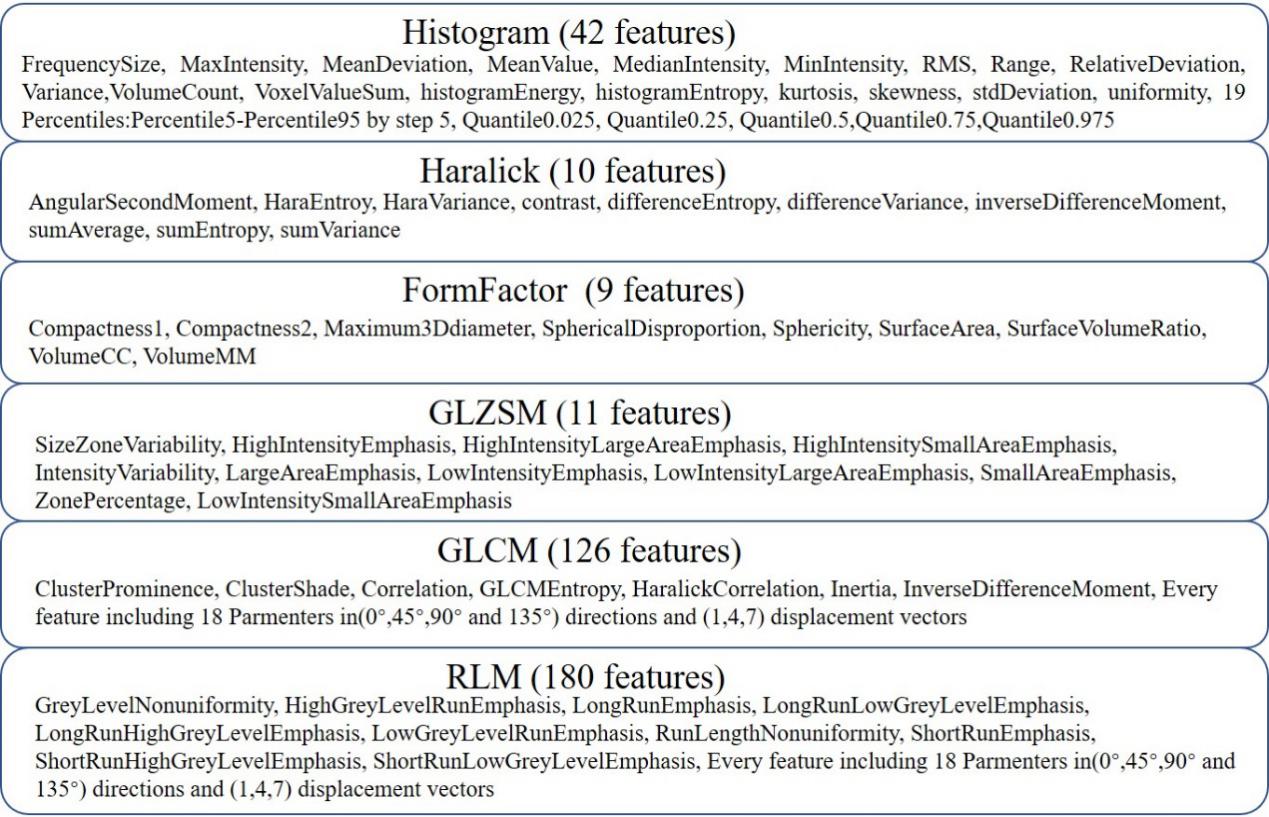


1. ANOVA + Mann-Whitney U test: Texture features with statistically significant for clinical outcome were extracted by analysis of variance and non-parametric test. Clinical outcome refers to progression of WMH or not. The AGK software will select the appropriate statistical method according to whether the data is a normal distribution.

2. Correlation analysis: This step involves calculating correlations between different features (Correlation_xx), and the correlation between features and clinical outcomes (Correlation_xy), to further remove redundant texture features.

3. Gradient boosting decision tree (GBDT): it was used to reduce the dimension of the remaining features. GBDT is an algorithm that classifies or regresses data by the linear combination of basic functions and reduces the residual generated in the training process.

**Details in dimension reduction of ROI WMHp**

1. The method for selecting features: Variance

parameters setted: {'threshold': 1.0}

num of remained features: 168


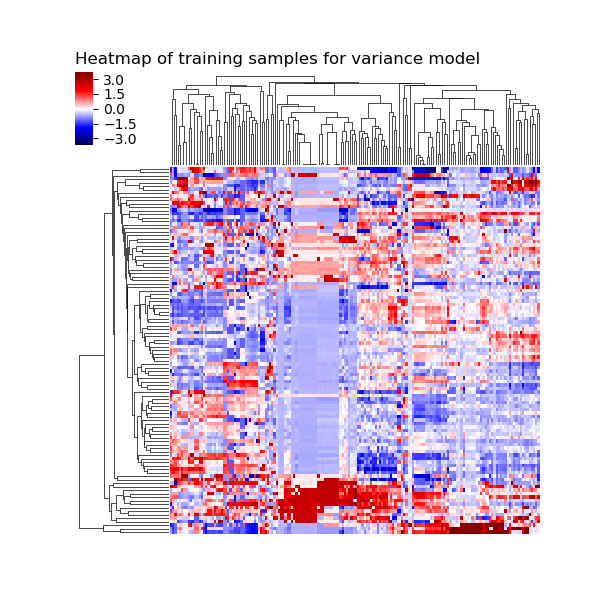

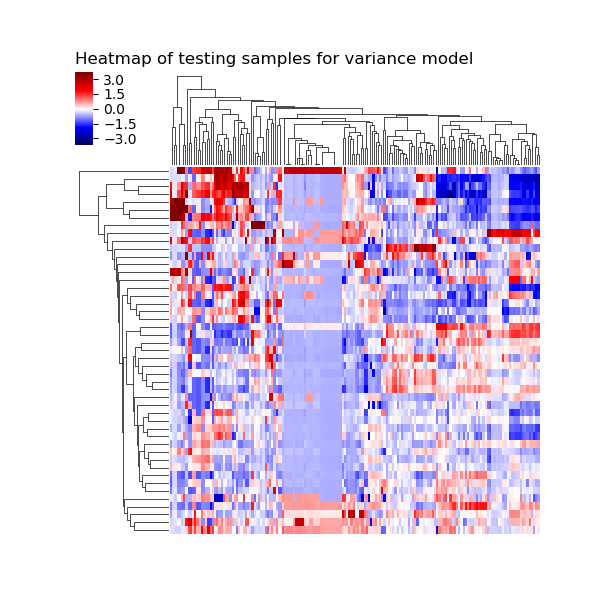


2. The method for selecting features: Correlation_xy

parameters setted: {'correlation': 0.1}

num of remained features: 40


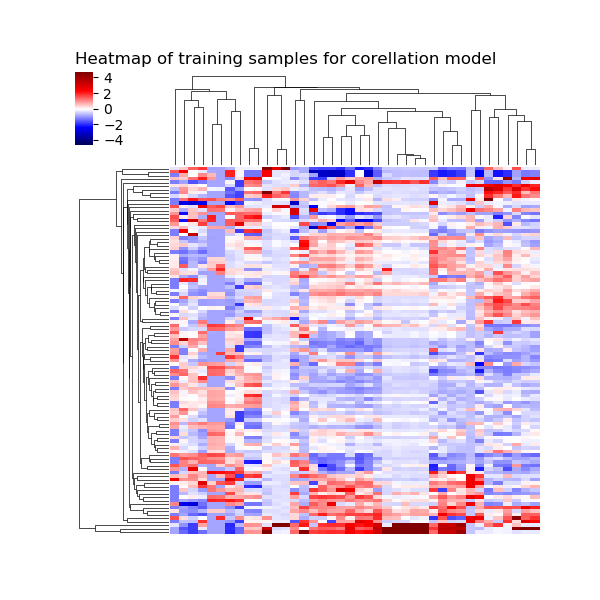

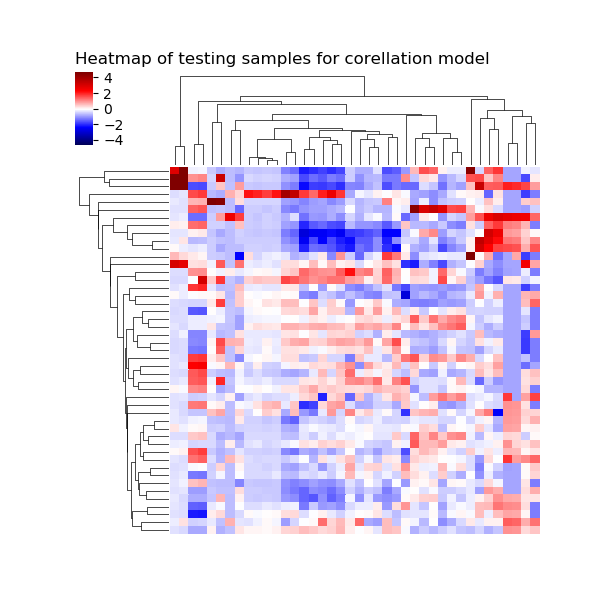


3. The method for selecting features: Correlation_xx

parameters setted: {'cutoff': 0.7}

num of remained features: 24


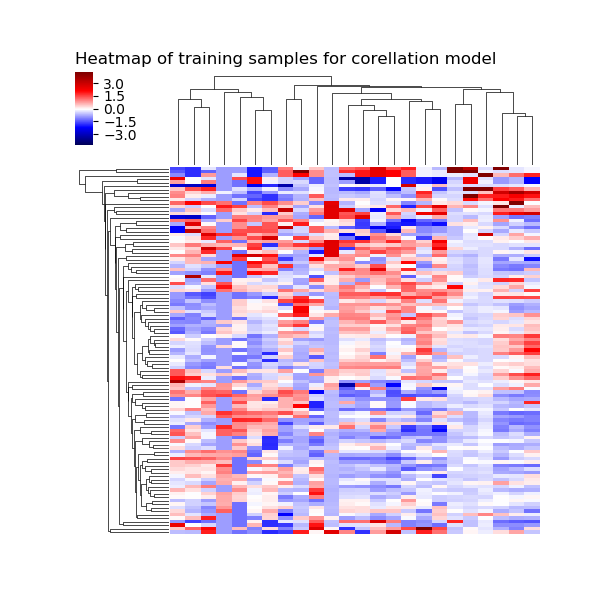

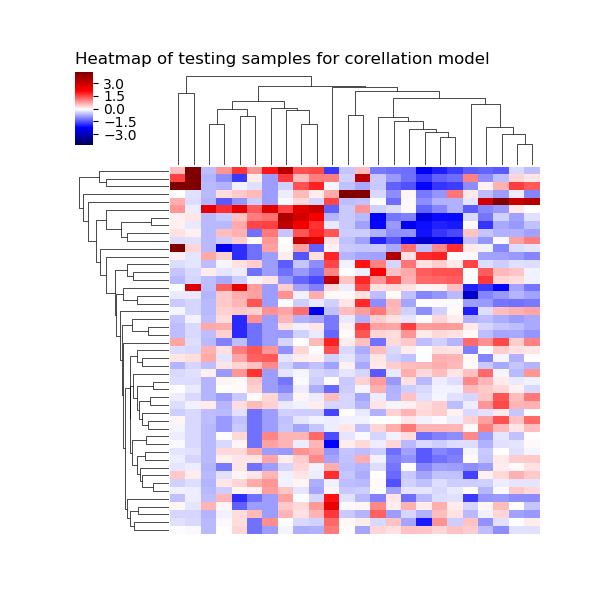


4. The method for selecting features: GBDT

parameters setted: {}

num of remained features: 12

remained features:

[['Quantile0.975']

['skewness']

['GLCMEntropy_AllDirection_offset4']

['Inertia_angle135_offset1']

['GreyLevelNonuniformity_AllDirection_offset7_SD']

['GreyLevelNonuniformity_angle0_offset1']

['LongRunEmphasis_AllDirection_offset4_SD']

['LongRunLowGreyLevelEmphasis_AllDirection_offset7']

['LowGreyLevelRunEmphasis_angle0_offset1']

['LowGreyLevelRunEmphasis_angle90_offset1']

['ShortRunEmphasis_angle0_offset7']

['ShortRunHighGreyLevelEmphasis_AllDirection_offset4_SD']]


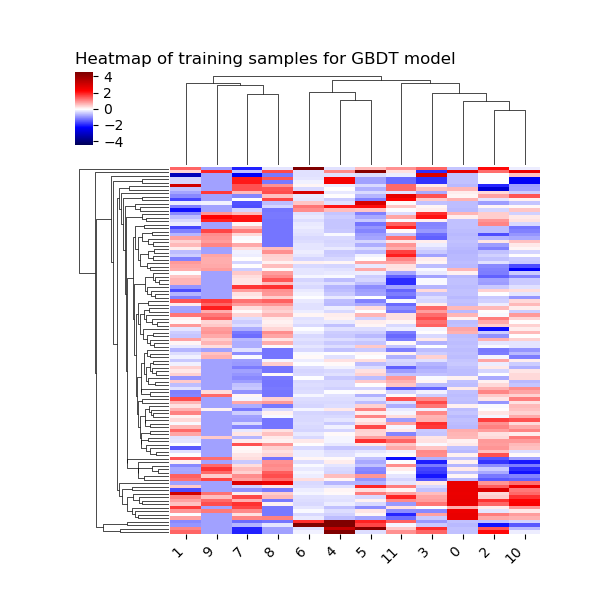

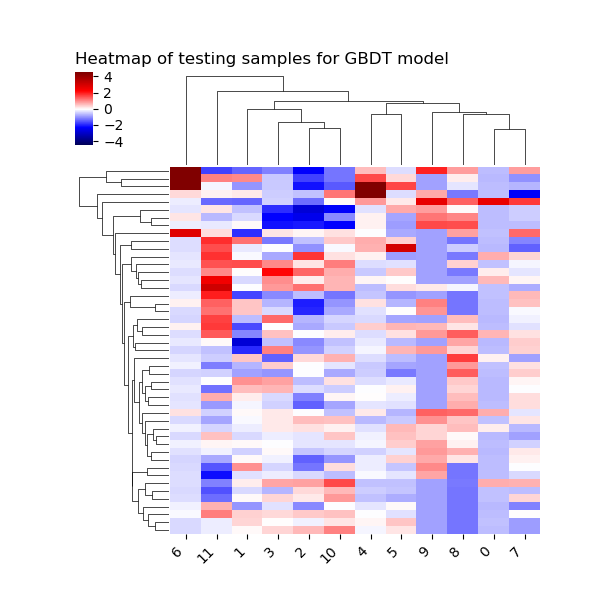


5. The best machine learning method: Logistic.

The rad-score of WMHp was calculated using the formula for the 12 features:

Radscore of WMHp= -0.68591172 +0.26959636* Inertia_angle135_offset1

+0.57706731* LowGreyLevelRunEmphasis_angle90_offset1

-0.2888143* LowGreyLevelRunEmphasis_angle0_offset1

+0.43968535* ShortRunHighGreyLevelEmphasis_AllDirection_offset4_SD

-0.67965934* LongRunLowGreyLevelEmphasis_AllDirection_offset7
-0.64322367* GreyLevelNonuniformity_angle0_offset1 +0.43103926* skewness

-0.4867927* LongRunEmphasis_AllDirection_offset4_SD

+0.32125272* GreyLevelNonuniformity_AllDirection_offset7_SD

-0.21215498* Quantile0.975 +0.17701112* ShortRunEmphasis_angle0_offset7
+0.07311699* GLCMEntropy_AllDirection_offset4

**Details in dimension reduction of ROI WBWM**

1. The method for selecting features: Variance

parameters setted: {'threshold': 1.0}
num of remained features: 172


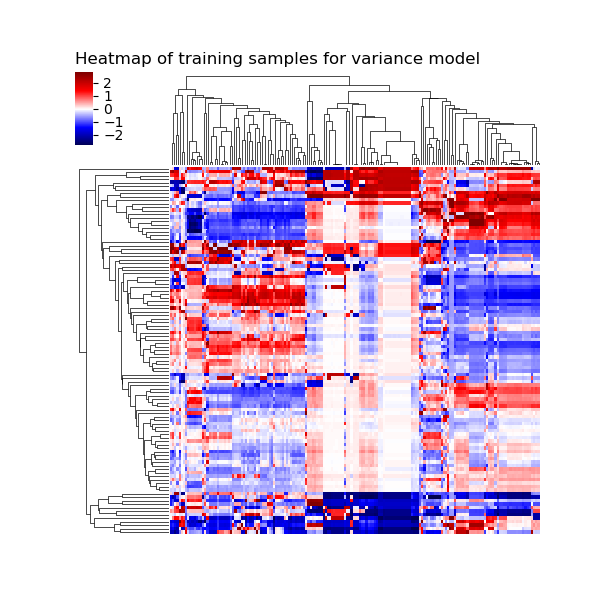

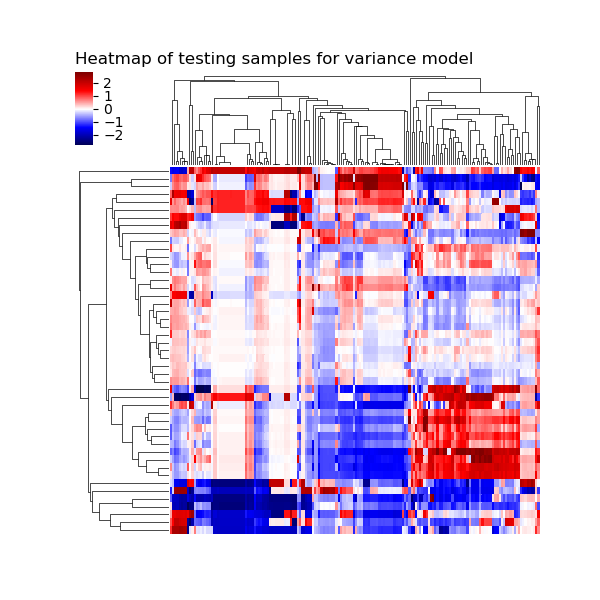


2. The method for selecting features: Correlation_xy

parameters setted: {'correlation': 0.1}
num of remained features: 104


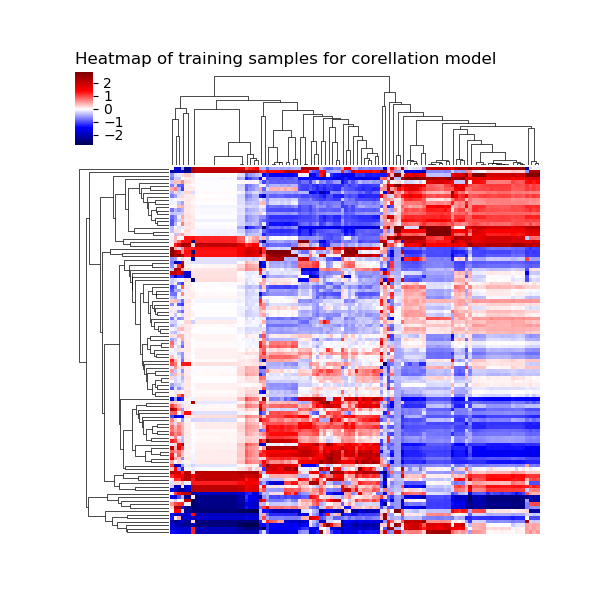

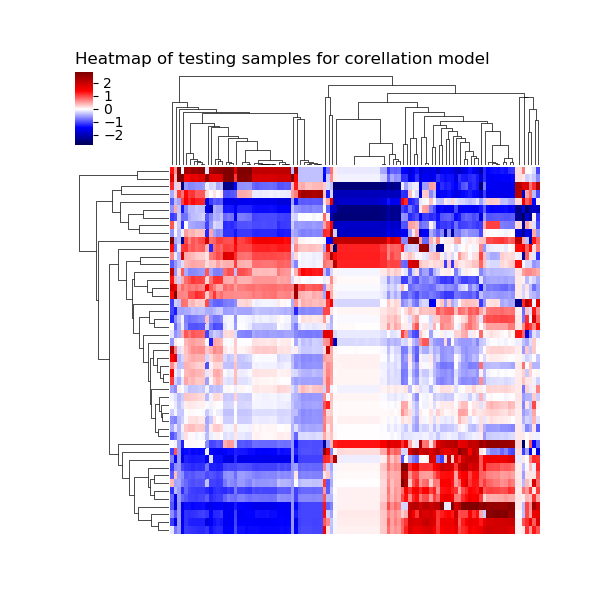


3. The method for selecting features: Correlation_xx

parameters setted: {'cutoff': 0.7}
num of remained features: 20


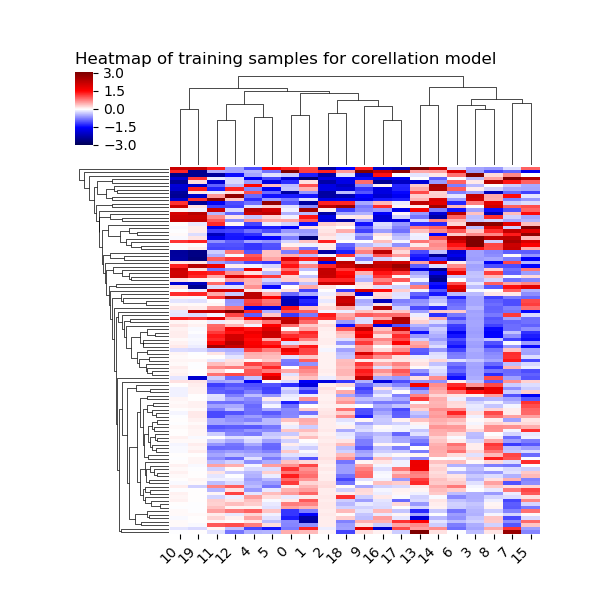

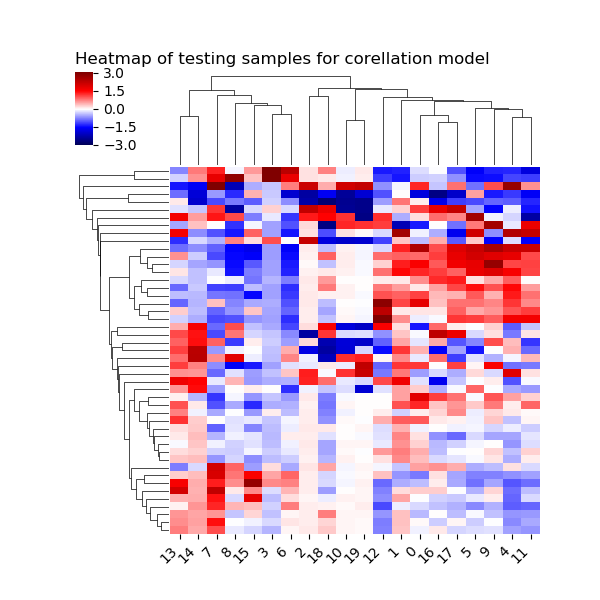


4. The method for selecting features: GBDT

parameters setted: {}
num of remained features: 7
remained features:
[['MeanDeviation']
 ['Percentile50']
 ['ClusterProminence_AllDirection_offset7_SD']
 ['Correlation_angle45_offset7']
 ['HaralickCorrelation_AllDirection_offset7_SD']
 ['ShortRunHighGreyLevelEmphasis_AllDirection_offset1_SD']
 ['ShortRunHighGreyLevelEmphasis_AllDirection_offset7_SD']]


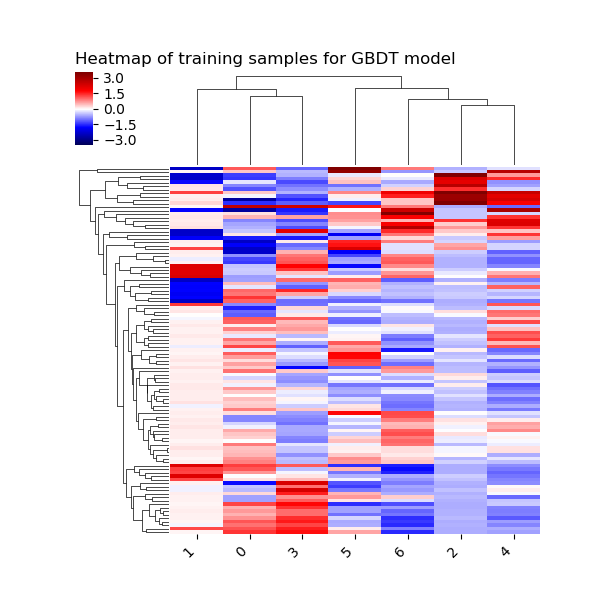

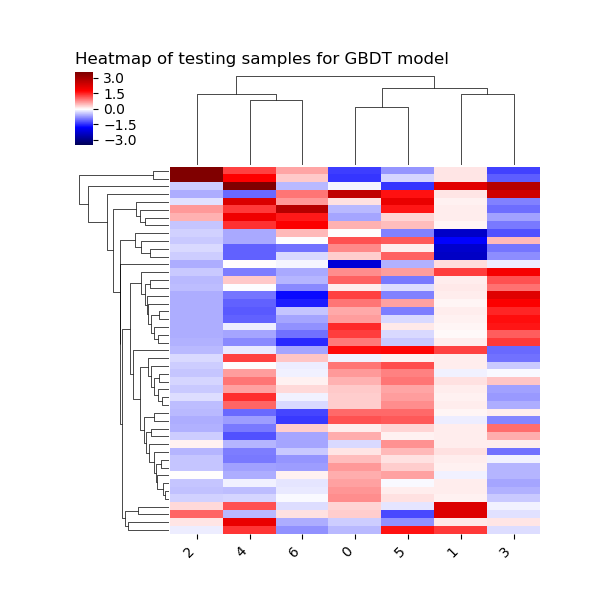


**5. The best machine learning method: Logistic**

The rad-score of WBWM was calculated using the formula for the 7 features:

Radscore of WBWM= -0.59882315

+0.65245291* ShortRunHighGreyLevelEmphasis_AllDirection_offset7_SD

+0.43563889* ClusterProminence_AllDirection_offset7_SD

-0.36482918* Percentile50 +0.28989747* Correlation_angle45_offset7

+0.23085487* ShortRunHighGreyLevelEmphasis_AllDirection_offset1_SD
-0.1083734* MeanDeviation +0.06721037 HaralickCorrelation_AllDirection_offset7_SD
